# The Effect of Pedal Peptide-Type Neuropeptide on Locomotor Behavior and Muscle Physiology in the Sea Cucumber *Apostichopus japonicus*

**DOI:** 10.3389/fphys.2020.559348

**Published:** 2020-10-22

**Authors:** Kui Ding, Libin Zhang, Xinhao Fan, Xueying Guo, Xiang Liu, Hongsheng Yang

**Affiliations:** ^1^CAS Key Laboratory of Marine Ecology and Environmental Sciences, Institute of Oceanology, Chinese Academy of Sciences, Qingdao, China; ^2^Laboratory for Marine Ecology and Environmental Science, Qingdao National Laboratory for Marine Science and Technology, Qingdao, China; ^3^Center for Ocean Mega-Science, Chinese Academy of Sciences, Qingdao, China; ^4^CAS Engineering Laboratory for Marine Ranching, Institute of Oceanology, Chinese Academy of Sciences, Qingdao, China; ^5^University of Chinese Academy of Sciences, Beijing, China; ^6^State Key Laboratory of Satellite Ocean Environment Dynamics, Second Institute of Oceanography, Ministry of Natural Resources, Hangzhou, China; ^7^The Innovation Academy of Seed Design, Chinese Academy of Sciences, Wuhan, China

**Keywords:** echinoderm, locomotor performance, muscle metabolite, pedal peptide, neuropeptide

## Abstract

Neuropeptides are endogenous active substances that are present in nervous tissues and participate in behavioral and physiological processes of the animal system. Locomotor behavior is basic to predation, escape, reproduction in animals, and neuropeptides play an important role in locomotion. In this study, the function of pedal peptide-type neuropeptide (PDP) in the process of locomotor behavior of the sea cucumber *Apostichopus japonicus* was evaluated. The locomotor behavior of *A. japonicus* was recorded by infrared camera before and after PDP administration, and muscle physiology was studied by ultra performance liquid chromatography and quadrupole time-off-light mass spectrometry (UPLC-Q-TOF-MS) to clarify the potential physiological mechanisms. The results showed that PDP enhanced the cumulative duration of moving significantly at the 7th h after injection, and reduced the mean and maximum velocity by 16.90 and 14.22% in *A. japonicus*. The data of muscle metabolomics suggested that some significantly changed metabolites were related to locomotor behavior of sea cucumbers. The decreases of phosphatidylethanolamine (PE) and phosphatidylcholine (PC) might result in the increases of lysophosphatidylcholines (lysoPC) and lysophosphatidylethanolamine (lysoPE), and suggested the change of fluidity and permeability in the muscle cell membrane, which would affect the physiology and function of muscle cells, and finally alter the locomotor behavior. In addition, the increased level of arachidonic acid (ARA) might activate K^+^ ion channels and then affect the signaling of muscle cells, or promote the sensitivity of muscle cells to Ca^2+^ and then result in the contractility of longitudinal muscles in sea cucumbers. ARA was also involved in the linoleic acid metabolism which was the only pathway that disturbed significantly after PDP administration. In conclusion, PDP participated in the regulation of locomotor behavior in the sea cucumber, and the decreased PE and PC, increased lysoPC, lysoPE and ARA might be the potential physiological mechanisms that responsible for behavioral effects of PDP in *A. japonicus*.

## Introduction

Neuropeptides generally refer to endogenous active substances that are present in nervous tissues and participate in the functions of animal nervous system. They are characterized by low contents, high activities, extensive functions and complex mechanisms. In evolution, neuropeptides are ancient neuronal signaling molecules that play a key role in the regulation of various physiological processes (homeostasis, energy metabolism) and behaviors (locomotion, feeding, and reproduction). Locomotor behavior is basic to predation, escape, habitat dominance, reproduction in animals, and a number of studies were focused on the functions and mechanisms of neuropeptides in locomotion (He et al., 2013; Pauls et al., 2019). Although the locomotor behavior of *A. japonicus* was studied thoroughly before (Pan et al., 2015), the mechanisms underlying locomotor behavior in this echinoderm species are still unclear. With the decoding of whole-genome and the application of transcriptomics and proteomics in *A. japonicus*, the neuropeptides in *A. japonicus* were recently identified (Chen et al., 2019), which provide the foundation to study the functions of neuropeptides in locomotor behavior in this species.

Pedal neuropeptides and orcokinin-type (PP/OK) neuropeptides are two structurally related neuropeptides that belong to the family of bilateral symmetric animal neuropeptides (Rowe and Elphick, 2012; Jekely, 2013). These two neuropeptides have been identified in many animals, including protostomia (such as nematodes and annelids) and deuterostome (such as echinoderms). Pedal peptide (PP) was originally found in the mollusk sea hare *Aplysia californica* and was predominately synthesized in the pedal ganglia of this species (Lloyd and Connolly, 1989). In particular, PP could cause pedal muscle contraction (Hall and Lloyd, 1990) and foot-related ciliary oscillation (Longley and Peterman, 2013), which indicated that it might play a role in the locomotor behavior of sea hares. In addition, orcokinin (OK) neuropeptides were firstly isolated from the crayfish *Orconectes limosus* nerve extract (Stangier et al., 1992), and OK-type neuropeptides could influence the circadian activity rhythm in *Leucophaea maderae* (Hofer and Homberg, 2006; Soehler et al., 2011; Wei and Stengl, 2011).

In recent years, studies on the PP/OK neuropeptides in echinoderms have been carried out widely. The echinoderm PP/OK neuropeptide was first discovered by analyzing the transcriptome data of the sea urchin *Strongylocentrotus purpuratus* (Rowe and Elphick, 2012). Subsequently, it was also demonstrated to be present in the sea cucumber (Rowe et al., 2014). Besides, a kind of muscle relaxant (SMP) was proved to be a PP/OK neuropeptide in the starfish *Patina pectinifera* (Kim et al., 2016). Five neuropeptides (ArPPLN1a-e) were identified in the SMP precursor (PP-type neuropeptide precursor 1; ArPPLNP1) of the starfish *Asterias rubens*, and ArPPLNP1 and neuropeptides in this precursor were widely expressed in sacral nerve cord, nerve ring, digestive system (such as cardiac stomach), body wall muscle and appendages (such as tube feet and spines) (Lin et al., 2017). This study showed that PP neuropeptides were present in the lateral motor nerves and the nerves that innervate the internal muscles, and caused relaxation of the body wall muscles, tube feet and cardiac stomach in this species (Lin et al., 2017). In addition, the distribution of the second PP/OK neuropeptide (ArPPLNP2) is extremely broad in the tissues of the starfish, and it can efficiently cause relaxation of the cardiac stomach (Lin et al., 2018).

Metabolomics is an important tool in systems biology research, which can detect the concentration of endogenous small molecules in tissues and shows the changes of metabolites’ concentrations in organisms under specific physiological conditions (Nicholson et al., 1999; Sun et al., 2017). The techniques of metabolomics include high performance liquid chromatography (HPLC) (Onchoi et al., 2008), gas chromatography-mass spectrometry (GC-MS) (Plumb et al., 2003), liquid chromatography-mass spectrometry (LC- MS) (Luo et al., 2007) and Nuclear Magnetic Resonance (NMR) (Robertson et al., 2000; Ji et al., 2013). Among them, ultra performance liquid chromatography and quadrupole time-off-light mass spectrometry (UPL-Q-TOF-MS) have the features of high resolution and high sensitivity, which can detect changes in differential metabolites in biological fluids or tissues quickly and effectively (Wilson et al., 2005; O’Connor and Mortishire-Smith, 2006). At present, this technology has been successfully applied to evaluate the metabolic physiology of sea cucumber muscle in breeding and non-propagation stages (Ru et al., 2017), and to study the effects of melatonin on muscle physiology in sea cucumbers (Ding et al., 2019).

The sea cucumber *A. japonicus* is the most important commercial species in echinoderms, and it is widely distributed along the coasts of north-west Pacific Ocean (35°N – 44°N) (Yang et al., 2015). To date, this species is cultured extensively in China. According to the *China Fishery Statistical Yearbook 2020*, the total marine aquaculture area and yield of sea cucumbers reached 246,745 ha and 171,700 tons in 2019. In present study, *A. japonicus* was used as a model system to study the function of PDP in the process of locomotor behavior. A pedal peptide-type neuropeptide (C-terminal serine is amidated, as determined from mass spec data.) from the Ajnp7 precursor protein (Rowe and Elphick, 2012; Chen et al., 2019) was synthesized artificially by biological techniques. The EthoVision XT software was used to analysis the changes of locomotor behavior in *A. japonicus* after PDP administration. In addition, the key metabolites and pathways are identified by UPL-Q-TOF-MS metabolomics to clarify the potential mechanisms underlying the effect of PDP on locomotor behavior.

## Materials and Methods

### The Synthesis of PDP

The PDP sequence was derived from the PP/OK neuropeptide precursor protein obtained by Rowe et al. (2014) by analyzing the transcriptome data of the sea cucumber. The C-terminal serine amidation of the FGSSQIMDPLRYSLVS sequence was finally determined by mass spec data (Chen et al., 2019). Pedal peptide-type neuropeptide was synthesized by GL Biochem (Shanghai) Peptide Ltd., using peptide solid phase syntheses, and the molecular formula is C_80_H_127_N_21_O_24_S, molecular mass is 1790.09 g/mol and purity is 99.80%. The product of PDP was stored at −20°C until use.

### Animals and Maintenance

The sea cucumbers were collected from the outdoor aquaculture pond in Zhuwang Port, Laizhou, Yantai (37°15.656′N, 119°53.985′E). After taking sea cucumbers back to the Qingdao laboratory, they were placed in a tank that was prepared in advance. The tank had a capacity of 1,500L, and was filled with filtered seawater and oxygenated by an air pump (dissolved oxygen >6.0 mg/L), the water temperature was maintained at 15 ± 0.5°C, the salinity was 30 ppt, and the pH was 8.0. Sea cucumbers were fed by the self-made diet (70% sea mud and 30% Sargassum powder), and food debris and feces were removed every morning, then half of the water was replaced once a day at 8 o’clock. The holding time lasts for 2 weeks.

### PDP Administration, Locomotor Behavior Tests and Behavioral Data Analysis

Twenty-four healthy sea cucumbers were selected from the holding tank for behavioral experiments. They were randomly divided into 2 groups (98.4 ± 16.2g, *n* = 12 sea cucumber/group), one of them was set as PDP group, and sea cucumbers in this group were injected with 0.1% (v/w) 10^–5^ M PDP solution into their coeloms (the PDP was dissolved in 1xPBS solution and diluted to 10^–5^ M by sterilized sea water) (Kato et al., 2009). The other one was the CON group, and sea cucumbers were injected with an equal amount of 1xPBS solution. The locomotor behavior of each individual sea cucumber was recorded separately in one glass tank (50 × 50 × 50 cm), and 24 identical tanks were used in this study. A white acrylic plate was placed at the bottom of the glass tank to facilitate the movement tracking with EthoVision XT (version 10.1) software (Noldus Inc., Netherlands). The water level in the glass tank was about 30 cm. An infrared camera (Hikvision DS-2CD3310D-I, 4MM, Hangzhou) was fixed by a plastic bracket above each tank. In order to ensure the uniformity of the experimental conditions, the experiment was carried out under full dark conditions. Before the experiment, the individual sea cucumber was placed in each tank for 24 h to familiarize the experimental environment (Ding et al., 2019). After that, all sea cucumbers in the tank were recorded for 3 h; then, twelve of them were injected with PDP solution, others were administrated by the 1xPBS solution. The injections were performed under dim red light with a 1 mL ICO syringe (JIANSHI^®^, Luohe, China) and a 0.3 mm microneedle (Pinillos et al., 2001).

The Hikvision infrared camera was used to record the locomotor behavior of each sea cucumber for 12 h (3 h before injection, 9 h after injection). The videos of locomotor behavior were analyzed by EthoVision software to quantify the locomotor behavior, and the behavioral indicators, including total distance traveled, cumulative duration of movement, mean and maximum velocity, were obtained. Continuous alternation of body contraction and relaxation makes the sea cucumber move ahead (Pan et al., 2015). Therefore, we defined one contraction and relaxation of body as one step, and the moving distance of one step is the stride length. The number of movement steps was also counted, and average stride length, stride frequency, and stride velocity were calculated in this study.

One-way analysis of variance and Tukey’s *post hoc* multiple comparison tests (SPSS 20.0 software) were used to analyze the data of sea cucumber locomotor behavior. A probability level of *p* < 0.05 was considered to be statistically significant.

### Muscle Sample Collection, UPLC-Q-TOF-MS Detection and Statistical Analysis

The behavioral data indicating that the time point of the significant difference of locomotor behavior between the treatment group and control group was the 7th h after PDP injection. 48 healthy sea cucumbers were selected from the holding water tanks and randomly divided into 2 groups (*n* = 24). The control group (CON) and the administrated group (PDP) were treated in the same way of behavioral study, respectively. About 2 g of longitudinal muscle tissue was sheared off from each sea cucumber at the 7th h after administration, and was washed with ultrapure water, dried with absorbent paper, placed in a sterile tube and stored in a refrigerator at −80°C.

Every 3 samples in both groups were combined in 1 tube for metabolomic detection. UPLC-Q-TOF-MS method was used in this study, and the detailed steps and statistical analysis were the same as previous research (Ding et al., 2019).

## Results

### Effect of PDP on Locomotor Behavior of *A. japonicus*

**FIGURE 1:**
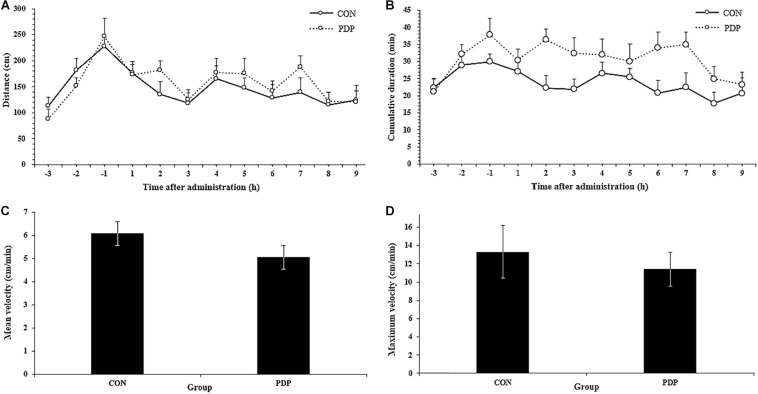
Total distance traveled per hour **(A)**, cumulative duration of movement per hour **(B)**, and mean and maximum velocity **(C,D)** for *A. japonicus* in the control (CON) and pedal peptide-type neuropeptide injected (PDP) groups. Each symbol or bar and vertical line represents the mean ± SEM (*N* = 12, *p* < 0.05).

In Figure 1, the results of EthoVision software analysis showed the total moving distance, cumulative duration of moving, average and maximum velocity from CON and PDP groups. The distances of sea cucumbers moved per hour ranged from 115.05 to 176.84 cm in the CON group and 120.57 to 188.31 cm in the PDP group, and they increased to some extent after PDP administration, but were not statistically significant (Figure 1A; *F* = 7.235, *p* > 0.05). The average total distance moved per hour after injection was 12.50 ± 1.29 m in the CON group and 14.05 ± 1.85 m in the PDP group. However, the cumulative duration of moving within each hour increased in the PDP group, and the significant difference between CON and PDP groups occurred at 7 h after injection (Figure 1B; *F* = 10.364, *p* < 0.05). The hourly duration of moving was between 17.81 and 27.15 min in the CON group, while in the PDP group, it was between 23.32 and 36.35 min (Figure 1B). The total cumulative duration of moving after injection was 205.30 ± 19.35 min (CON group) and 278.67 ± 24.63 min (PDP group). Moreover, the mean and maximum velocities of the PDP group were 16.90 and 14.22% lower than that of the CON group, but they were not statistically significant (Figures 1C,D; *p* > 0.05).

**FIGURE 2:**
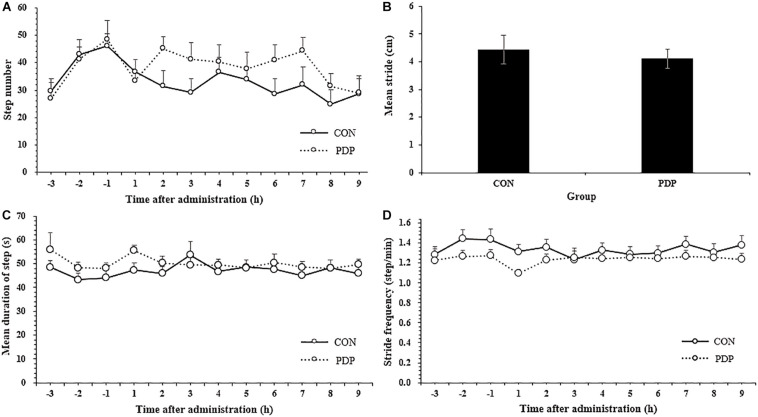
Average total number of steps taken per hour **(A)**, stride **(B)**, stride frequency **(C)**, and stride velocity **(D)** of *A. japonicus* in the control (CON) and pedal peptide-type neuropeptide injected (PDP) groups. Each vertical line represents the mean ± SEM (*N* = 12, *p* < 0.05).

Figure 2 shows results of other measures of locomotor performance, including the number of steps moving, average stride, mean duration of each step and step velocity in each hour. The average number of steps moving within each hour ranged from 24.83 to 36.67 in the control and 29.08 to 45.08 in the treatment, and almost all of them in the PDP group were higher than that in the CON group, despite none of them were statistically significant (Figure 2A; *F* = 7.869, *p* > 0.05). The number of total steps taken after injection was 281.58 ± 23.49 in the CON group and 343.67 ± 26.58 in the PDP group. In addition, the average stride in the PDP group decreased to some extent, but it was no statistically significant, neither the mean duration of steps taken and stride frequency (Figures 2B–D).

### Effect of PDP on Muscle Physiology of *A. japonicus*

**FIGURE 3:**
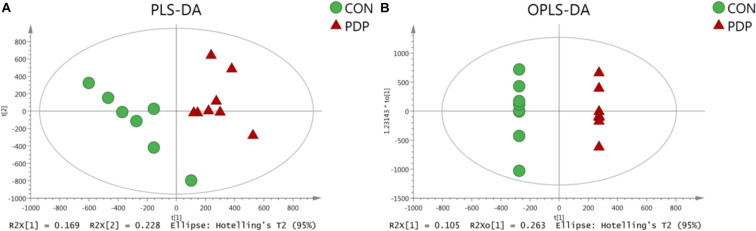
The PLS-DA **(A)** and OPLS-DA **(B)** scores plot of muscle metabolites from the control (CON) and pedal peptide-type neuropeptide injected (PDP) groups. The abscissa and ordinate represent the first principal component (PC1) and the second principal component (PC2), respectively.

**FIGURE 4:**
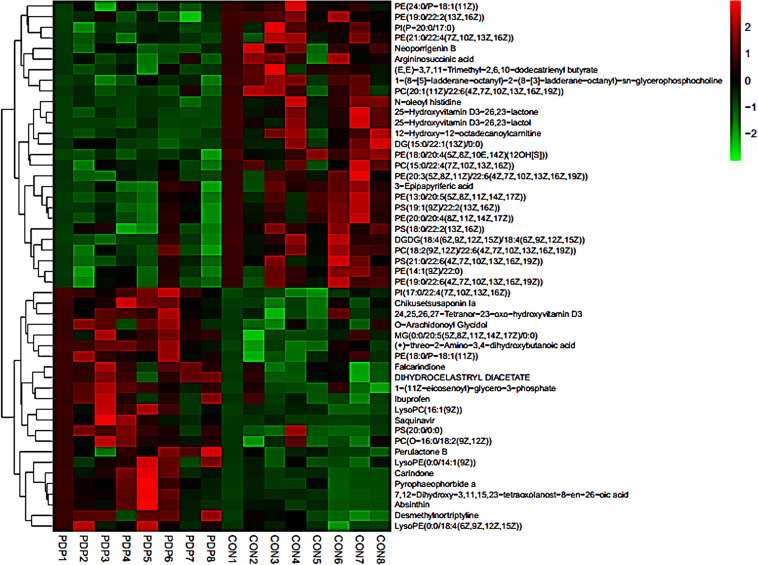
The heat maps of overall differential metabolites from the control (CON) and pedal peptide-type neuropeptide injected (PDP) groups. Each line represents a differential metabolite and each cross represents a muscle sample. Different colors represent different higher abundance intensity (mean value acquired from all detected samples of the same group).

**FIGURE 5:**
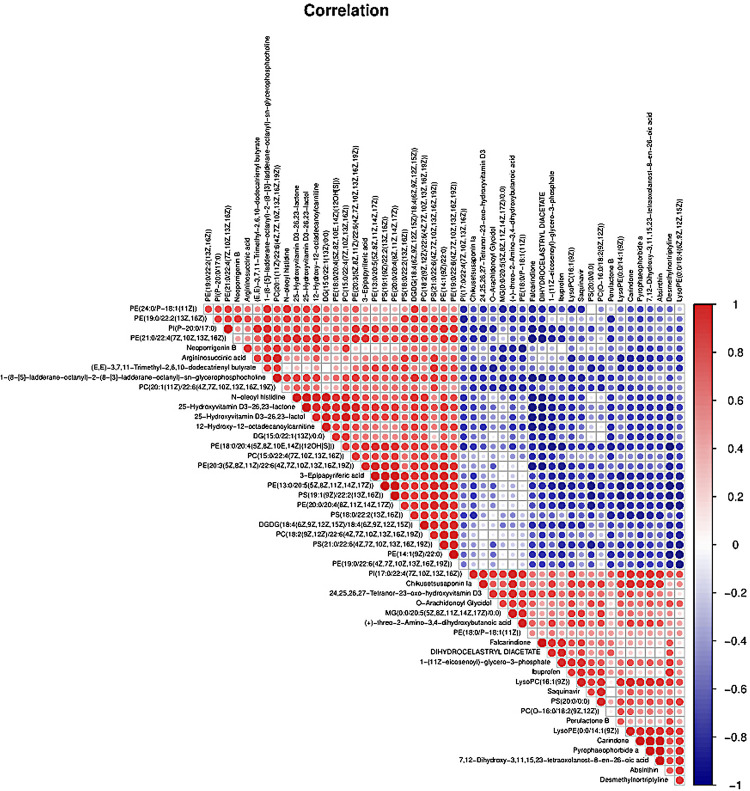
The correlation analysis of overall differential metabolites from the control (CON) and pedal peptide-type neuropeptide injected (PDP) groups. The color of each line represents the Pearson correlation coefficient of two differential metabolites. Red for positive correlation and blue for negative correlation.

PLS-DA and OPLS-DA were used to identify the metabolic alterations of muscle tissues between CON and PDP groups. In the PLS-DA plot, the abscissa represents the first principal component PC1 (t[1]), and the ordinate represents the second principal component PC2 (t[2]) (Figure 3A). While in the OPLS-DA plot, the abscissa represents the predictive principal component, and the ordinate represents the orthogonal principal component (Figure 3B). Each spot in the figure represents one sample. Both plots reveal that the CON (green spot) group and PDP (red spot) group are clearly separated from each other. Besides, Figure 4 shows the heat map of overall differential metabolites from CON and PDP groups. Each transverse line represents a differential metabolite and each cross represents a muscle sample. Different colors represent different higher abundance intensity (mean value acquired from all detected samples of the same group). The correlation analysis of overall differential metabolites from CON and PDP groups is shown in Figure 5. The color of each dot represents the Pearson’s correlation coefficient of two differential metabolites. Red for positive correlation and blue for negative correlation.

**FIGURE 6:**
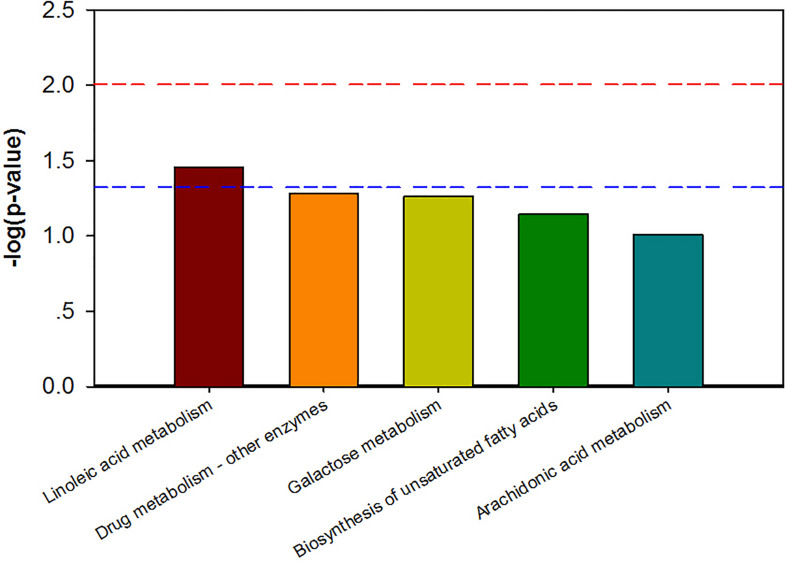
The KEGG pathway enrichment of overall differential metabolites from the control (CON) and pedal peptide-type neuropeptide injected (PDP) groups. Horizontal axis for enriched pathway; vertical axis for the significance level of pathway enrichment. Above the red and blue dashed lines represents *p* < 0.01 and *p* < 0.05.

Combining *t*-test (*p* < 0.05) and OPLS-DA model (VIP > 1.0) results, 31 key differential metabolites between CON and PDP groups were identified, and 15 of them were positive ion patterns and 16 of them were negative ion patterns (Table 1). Twenty-two metabolites such as piperidine, phosphatidylethanolamine (PE), phosphatidylcholine (PC), neoporrigenin B, and L-3-Aminodihydro-2(3H)-furanone decreased significantly, and 9 metabolites such as lysophosphatidylethanolamine (LysoPE), lysophosphatidylcholine (LysoPC), carindone, and arachidonic acid increased significantly in the PDP group. Correlation analysis of 50 differential metabolites illustrated that the correlation between LysoPC and PC were negative, as well as LysoPE and PE (Figure 5). Metabolic pathway enrichment analysis indicated that these differential metabolites were involved linoleic acid metabolism, drug metabolism – other enzymes, galactose metabolism, biosynthesis of unsaturated fatty acids and arachidonic acid metabolism, in which the linoleic acid metabolism pathway was disturbed significantly after PDP administration (Figure 6, *p* < 0.05). The dataset of metabolomics in this study was uploaded in figshare (https://doi.org/10.6084/m9.figshare.12436949.v1).

**TABLE 1:** Muscle metabolites with concentrations that differed significantly between the control (CON) and pedal peptide-type neuropeptide injected (PDP) groups, including the ion mode [positive (pos) or negative (neg)], mass (compound molecular weight), RT [retention time (min)], VIP (variable importance in the projection), FC (fold change, PDP/CON) and *p* value of these metabolites.

Metabolite	Ion mode	Mass (Da)	RT (min)	VIP	FC_(PDP/CON)_	*p*
Piperidine	pos	86.097	2.044	1.486	0.502	0.026
PE[24:0/P-18:1(11Z)]	neg	858.620	11.371	1.594	0.653	0.005
PE[21:0/22:4(7Z,10Z,13Z,16Z])	pos	860.616	10.740	6.856	0.765	0.006
PE[20:3(5Z,8Z,11Z)/22:6(4Z,7Z,10Z,13Z,16Z,19Z)]	neg	812.524	11.434	1.431	0.358	0.012
PE[20:1(11Z)/22:6(4Z,7Z,10Z,13Z,16Z,19Z)]	neg	818.560	14.004	2.392	0.781	0.016
PE[20:0/20:4(8Z,11Z,14Z,17Z)]	neg	776.559	14.004	6.457	0.870	0.006
PE[19:0/22:6(4Z,7Z,10Z,13Z,16Z,19Z)]	neg	850.562	14.004	2.883	0.704	0.009
PE[19:0/22:2(13Z,16Z)]	neg	858.621	10.987	3.734	0.804	0.001
PE[18:0/20:4(5Z,8Z,10E,14Z) (12OH[S])]	pos	766.535	13.831	2.235	0.525	0.000
PE[14:1(9Z)/22:0]	neg	790.541	14.004	5.754	0.695	0.004
PE[13:0/20:5(5Z,8Z,11Z,14Z,17Z)]	neg	694.445	14.004	1.353	0.844	0.003
PC[20:2(11Z,14Z)/22:6(4Z,7Z,10Z,13Z,16Z,19Z)]	pos	840.588	13.999	5.361	0.805	0.018
PC[20:1(11Z)/22:6(4Z,7Z,10Z,13Z,16Z,19Z)]	neg	904.605	10.884	1.083	0.442	0.009
PC[19:0/0:0]	neg	536.372	9.195	1.034	0.469	0.023
PC[18:2(9Z,12Z)/22:6(4Z,7Z,10Z,13Z,16Z,19Z)]	pos	830.567	13.999	6.632	0.694	0.006
PC[18:1(11Z)/22:6(4Z,7Z,10Z,13Z,16Z,19Z)]	pos	814.572	13.999	12.684	0.853	0.017
PC[18:0/18:2(6Z,9Z)]	pos	808.585	14.012	7.948	0.780	0.037
PC[15:0/22:4(7Z,10Z,13Z,16Z)]	pos	796.585	13.759	3.595	0.569	0.012
O-Arachidonoyl Glycidol	pos	361.273	5.962	1.768	1.295	0.010
N-oleoyl histidine	pos	839.640	13.687	1.563	0.607	0.001
Neoporrigenin B	pos	464.337	5.241	1.088	0.442	0.007
N-arachidonoyl taurine	neg	410.237	6.757	2.925	0.458	0.023
Melibiose	pos	325.113	4.627	2.093	3.048	0.049
LysoPE[0:0/18:4(6Z,9Z,12Z,15Z)]	neg	518.282	2.668	1.148	1.694	0.012
LysoPE[0:0/14:1(9Z)]	neg	424.237	4.745	1.119	1.957	0.010
LysoPC[16:1(9Z)]	neg	538.315	5.838	1.163	3.843	0.003
L-3-Aminodihydro-2(3H)-furanone	pos	84.045	2.044	1.185	0.499	0.026
Falcarindione	pos	513.298	13.211	2.985	1.659	0.002
Carindone	neg	511.307	5.132	1.151	3.992	0.004
Arachidonic acid	neg	303.233	5.975	1.872	1.144	0.038
4-Deoxytetronic acid	pos	87.044	0.906	1.046	1.865	0.043

## Discussion

### Effect of PDP on Locomotor Performance of *A. japonicus*

The results of this study indicated that PDP was involved in the regulation of locomotor behavior of the sea cucumber *A. japonicus*, especially the locomotor endurance. To date, the effects of neuropeptides on animal locomotor behavior have been widely reported in mice, rats, locusts, fruit flies, sea hares, nematodes and other organisms (Pañeda et al., 2009; Kahsai et al., 2010; Yang et al., 2016; Hou et al., 2017; Pauls et al., 2019). Both Nocll neuropeptide and neuropeptide S (NPS) can stimulate the locomotor behavior in mice, and NPS induce this effect through corticotropin-releasing factor receptor 1 (Florin et al., 1997; Pañeda et al., 2009). Besides, neuropeptide F (NPF, including NPF1a, and NPF2) can suppress locomotor behavior, and NPS/nitric oxide pathway is essential for the plasticity of locomotor behavior during phase transition in swarming locust (Hou et al., 2017). GdFFD neuropeptide can significantly reduce the locomotor activity and induce a foot curl in the marine mollusc *Aplysia californica* (Yang et al., 2016). In drosophila, neuropeptide Drosophila tachykinin (DTK) participates in the regulation of spatial orientation, and the deficiency of DTK resulted in the decrease of locomotor behavior, while short neuropeptide F (sNPF) takes part in the fine regulation of locomotor performance (Kahsai et al., 2010). Sensory neurons trigger locomotor behavior by secreting neuropeptide (PDF-1) and glutamate, while neuropeptide (FLP-2) induces locomotor activity via an orexin-like receptor (FRPR-18) in the nematode *Caenorhabditis elegans* (Chen et al., 2016). Although the effects of different neuropeptides on locomotor behavior were widely studied in various animals, few studies focused on the pedal neuropeptide. Pedal neuropeptide can stimulate the feet muscle of *Aplysia* to increase the amplitude and relaxation rate of contractions driven by neuronal or intracellular stimulation of pedal motor neurons, and pedal neurons have the function of modulating foot muscle contractility during locomotor behavior in *Aplysia californica* (Hall and Lloyd, 1990). The results of this study indicated that the stride of sea cucumbers decreased to some extent after PDP injection, indicating that PDP might participate in the regulation of muscle contraction during locomotor activity. In addition, the distance moved, number of steps taken and cumulative duration of moving were increased after PDP administration, while the cumulative duration of moving in each hour was significantly higher in PDP group at the 7th h, indicating that PDP can enhance the locomotor endurance of sea cucumbers. The decrease of mean velocity, maximum velocity and mean step velocity, and the increase of average duration of moving, indicated that PDP may reduce the efficiency of locomotor activity in the sea cucumber to some extent.

In conclusion, PDP participated in the regulation of locomotor behavior in the sea cucumber *A. japonicus*, and more precisely, it could enhance the endurance of locomotion. This finding would provide evidence for the effect of PDP on the locomotor behavior of sea cucumbers.

### Potential Mechanisms Underlying the Effect of PDP on Locomotor Performance in the Sea Cucumber *A. japonicus*

In this study, the time point at which the significant differentiation of locomotor performance occurred between control and PDP administrated *A. japonicus* (the 7th h after injection) was selected to measure the changes of metabolite levels in the muscle tissues of these two groups. Our results revealed that the muscle metabolite profiles in sea cucumbers after PDP treatment were different from the control, and one metabolic pathway was disturbed significantly. PE and PC levels were significantly decreased in the muscle tissues of the PDP group, while the levels of LysoPE, LysoPC and ARA increased significantly. In addition, ARA was involved in the pathway of linoleic acid metabolism according to KEGG. These metabolites and linoleic acid metabolic pathways are likely to be potential mechanisms that underlying the effect of PDP on locomotor endurance and efficiency in *A. japonicus*.

In muscles, the composition of different phospholipids and phosphatidylglycerols types is closely related to cell membrane fluidity, lipid rafts, membrane protein dynamics and insulin receptor dynamics (Pilch et al., 1980; Nadiv et al., 1994; Gorski et al., 1999; Saha et al., 2016). Phosphatidylethanolamine (PE) and phosphatidylcholine (PC) are the major phospholipids in cell membrane, and PE accounts for 20–30% of the total phospholipid pool, while PC accounts for about 0.5% (Takagi, 1971). Knocking out PC and PE-related specific enzymes in model animal resulted in the decrease of PE synthesis, increase of PC:PE value, reduced skeletal muscle, declined activity of endoplasmic reticulum/sarcoplasmic reticulum (ER/SR) Ca^2+^ ATPase (SERCA), and finally decreased locomotor performance (Funai et al., 2013; Selathurai et al., 2015; Funai et al., 2016). Therefore, skeletal muscle growth, locomotor performance and glucose metabolism are likely to be related with the value of PC: PE. Phospholipid composition is biologically important for the functions that related to mitochondria, cell growth, muscle contraction, locomotor performance, and insulin sensitivity in skeletal muscle (Heden et al., 2016). In addition, acute and long-term physical exercise can reduce the value of PC:PE in human skeletal muscle, and mitochondrial function is involved in the potential molecular correlation between PC: PE ratio and insulin sensitivity in skeletal muscle (Lee et al., 2018). Thus, PC: PE value play a critical role in metabolism and insulin sensitivity of skeletal muscle (Wilson et al., 1981; Stuart et al., 1988; Goodyear and Kahn, 1998). The results of this study showed that phosphatidylethanolamine (PE) and phosphatidylcholine (PC) were decreased significantly in the muscle of sea cucumbers after PDP administration, although the change of PC: PE ratio was not clear according to our results, both PE and PC were degraded in the muscle cell membrane of sea cucumbers, and the fluidity and permeability of the cell membrane were changed, which will affect the physiology and function of muscle cells. This shift plays an important role in regulating animal behavioral plasticity (Wu et al., 2012). Considering that phospholipid composition is crucial for the transformation of muscle contraction and locomotor behavior in animals, and the PDP participates in the regulation of muscle contractility during the locomotion of animal (Hall and Lloyd, 1990), the decreases of phosphatidylethanolamine (PE) and phosphatidylcholine (PC) were likely to be a potential physiological mechanism that underlying the effect of PDPs on locomotor performance in *A. japonicus*.

Both lysophosphatidylethanolamine (LysoPE) and lysophosphatidylcholine (LysoPC) are part of lysophospholipids, and lysophosphatidylcholine (LysoPC) is also used as a biomarker for the diagnosis of diabetes (Oresic et al., 2008). Muscle metabolite profiles of sea cucumbers during reproductive period indicated that oxidative damage caused by elevated LysoPC was a potential mechanism for the decreased locomotor endurance of sea cucumbers (Ru et al., 2017). Although the level of lysophosphatidylcholine (LysoPC) was increased after PDP administration, the LysoPE was also up-regulated simultaneously, which is different from the previous results of muscle metabolite profiles of reproductive sea cucumbers. Therefore, the increases of both LysoPC and LysoPE were likely to be responsible for the increased locomotor endurance of *A. japonicus* after PDP treatment. LysoPC and LysoPE were converted from PE and PC by phospholipase A2; thus, the decreases of PE and PC might be the reasons for the increases of LysoPC and LysoPE in this study.

Arachidonic acid is a kind of polyunsaturated ω-6 fatty acid, which acts as precursor for many bioactive lipid mediators, and plays an important role in muscle anabolism. Few studies were focused on ARA in echinoderms. The composition of lipids and fatty acids in egg and body wall of sea urchin *Diadema savignyi* revealed that ARA accounts for the highest proportion (>50%) in the polyunsaturated fatty acids (Kim et al., 2018). ARA is likely to be an important component in the cells of echinoderm. In the process of muscle recovery after acute training in humans, the intake of ARA may enhance muscle adaptability (Mitchell et al., 2018). In addition, ARA can be oxidatively metabolized by cytochrome P450 epoxidase, and transformed into four regioisomeric epoxy eicosatrienoic acids (5,6-; 8,9-; 11,12-; 14,15-EET). They have the function of vasodilation, and cytochrome P450 metabolites of ARA can activate K^+^ ion channels of vascular smooth muscle (Hu and Kim, 1993). Besides, ARA and other fatty acids can directly activate K^+^ ion channels in smooth muscle cells (Ordway et al., 1989). It is well known that K^+^ ion channels are closely related to cell signal transduction. Besides, ARA can inhibit the activity of myosin phosphatase, which makes smooth muscle to be more sensitive to Ca^2+^ (Gong et al., 1992), and the release of Ca^2+^ will activate smooth muscle for contraction. The increase of ARA in the PDP administrated group might activate K^+^ ion channels in muscle cells, thereby affecting the signaling of muscle cells. At the same time, the increased level of ARA was likely to promote the sensitivity of muscle cells to Ca^2+^, and resulted in the contractility of longitudinal muscles in sea cucumbers. Thus, the elevated ARA in muscle tissues might be the potential physiological mechanism for the function of PDP in muscle contraction during locomotor behavior of *A. japonicus*.

## Conclusion

This study showed that pedal peptide-type neuropeptide was involved in the regulation of locomotor behavior in *A. japonicus*. The prolonged duration of moving after PDP administration indicated that PDP enhanced the endurance of locomotion. The results of muscle metabolomic revealed that the decrease of PE and PC levels, and the increase of LysoPC, LysoPE, and ARA levels in muscle tissues after PDP treatment were the potential mechanisms that underlying the effects of PDP on locomotor behavior in *A. japonicus*.

## Data Availability Statement

The raw data supporting the conclusions of this article will be made available by the authors, without undue reservation.

## Author Contributions

KD designed and performed the research. LZ contributed new reagents and analytic tools. XF, XG, XL, and KD analyzed the data of locomotor behavior. KD wrote the manuscript. HY supervised the research. All authors contributed to the article and approved the submitted version.

## Conflict of Interest

The authors declare that the research was conducted in the absence of any commercial or financial relationships that could be construed as a potential conflict of interest.
